# Cancer vaccines and RNA therapeutics: exploring the impact of microRNAs

**DOI:** 10.1038/s41541-026-01425-9

**Published:** 2026-03-25

**Authors:** Miriam Valeria Rojas-Rojas, Ainhoa Muiños-García, Cristian Arriaga-Canon, Luis Alonso Herrera

**Affiliations:** 1 Laboratorio de Innovación y Medicina de Precisión, Núcleo A, Instituto Nacional de Medicina Genómica, Ciudad de México, México; 2 Escuela de Medicina y Ciencias de la Salud, Tecnológico de Monterrey, Ciudad de México, México; 3 Instituto Nacional de Ciencias Médicas y Nutrición Salvador Zubirán, Ciudad de México, México

**Keywords:** Biotechnology, Cancer, Drug discovery, Immunology

## Abstract

RNA-based therapies have been studied for decades, gaining global recognition with the success of the Pfizer-BioNTech mRNA vaccine. Despite ongoing challenges in standardization and optimization, RNA therapeutics have become central to precision medicine. This success has driven interest in oncology applications, particularly microRNAs (miRNAs), which regulate gene expression and cancer-related processes. This article reviews RNA-based cancer therapies, highlighting miRNA potential and clinical translation challenges.

## From the lab to the pandemic: the rising of mRNA

Messenger RNA (mRNA) is a single-stranded ribonucleic acid that, for a long time, was primarily regarded as a transient intermediary in the flow of genetic information, responsible for encoding functional proteins and polypeptides^1^. However, its relevance received unprecedented global attention during the SARS-CoV-2 pandemic. Despite its recent spotlight, research on mRNA dates to the early 2000s, when Katalin Kariko and Drew Weissman—later awarded the Nobel Prize—demonstrated that RNA isolated from mammalian and bacteria cells, as well as in vitro transcripts, triggered immune response through dendritic cell activation. Subsequently, they introduced nucleosides modifications, including m5C, m6A, and pseudouridine, which effectively suppressed this immunogenicity^2^. These breakthroughs were pivotal in enabling the development of RNA-based therapeutic and catalyzed further research, leading to innovations later developed by companies such as Pfizer-BioNTech and Moderna, fundamental during the COVID-19 crisis.

Beyond the success of this intervention, the potential of mRNA as a therapeutic tool did not initially emerge in the context of infectious diseases, but rather in oncology^3^. The first attempt to use an mRNA-based vaccine as a cancer therapy was conducted in 1995, when David Curiel and his team investigated the immune response to a polynucleotide vaccine targeting the carcinoembryonic antigen (CEA) in animal models^4^. In this pilot study, seven C57BL/6 mice received intramuscular injections of mRNA transcripts encoding CEA and were subsequently challenged with tumor cells expressing the same antigen. Three weeks after exposure, five of the seven vaccinated mice exhibited detectable anti-CEA antibodies, while no antibody response was observed in the control group^5^. These findings provided early proof-of-concept for mRNA vaccines as cancer immunotherapy and established a foundation for expanding this technology into other areas of medicine. This study highlighted several key aspects required to advance this strategy: the design of immunotherapies targeting specific tumor-associated antigens such as CEA; the optimization of delivery systems to ensure efficient and sustained mRNA expression in target cells; and the development of immunological approaches to overcome tumor-associated antigen tolerance, aiming to trigger effective anti-tumor immunity.

What began in 1995 as a groundbreaking concept has since evolved into interventions that have transformed global health, exemplified by the success against SARS-CoV-2. Nevertheless, applying mRNA in cancer therapy still presents major challenges—from enhancing its molecular stability to improving its ability to precisely direct the immune system against malignant cells. Overcoming these hurdles demands a deeper understanding of its mechanisms of action and the fine-tuning of its immunogenic potential.

## An innovative mechanism

The mechanism of action of mRNA vaccines relies on their ability to modulate protein expression within host cells, eliciting a targeted immune response against tumor cells. Upon administration, the synthetic mRNA is taken up by host cells and translated by the cellular machinery into polypeptides that encode tumor antigens^6^. These antigens, classified as tumor-specific antigens (TSAs) or tumor-associated antigens (TAAs), are subsequently captured by antigen-presenting cells (APCs), such as dendritic cells.

Following antigen processing, fragments are presented on the cell surface via major histocompatibility complexes (MHCs), enabling the activation of CD4⁺ helper T cells and CD8⁺ cytotoxic T lymphocytes. Activated CD8⁺ T cells mediate direct tumor cell lysis through the release of perforins and granzymes, while CD4⁺ T cells amplify the immune response through the secretion of proinflammatory cytokines^4^. In addition, these vaccines influence the tumor immune microenvironment by promoting dendritic cell activation, enhancing antigen presentation through MHC class I and II pathways, and inducing long-lasting immune memory^7^. Furthermore, mRNA vaccination promotes B-cell activation, leading to the generation of tumor-specific antibodies. These antibodies contribute to tumor clearance via mechanisms such as antibody-dependent cell-mediated cytotoxicity (ADCC) and activation of the complement cascade^8^.

In fact, the clinical application of this approach is evident in ongoing trials evaluating the efficacy and safety of mRNA vaccines in cancer immunotherapy. Although the number of registered studies remains limited, with fewer than 150 clinical trials, this field has experienced accelerated growth driven by advancements in mRNA stability, improved delivery systems such as lipid nanoparticles (LNPs), and an enhanced ability to elicit specific antitumor immune responses (Table 1).

**Table 1:** Current clinical landscape of RNA-based cancer therapies, including personalized vaccines and delivery strategies using lipid nanoparticles (LNPs) and other emerging platforms

ClinicalTrials.gov ID	Cancer type	RNA type	Clinical phase	Preliminar outcomes
NCT03480152: Messenger RNA (mRNA)-Based, Personalized Cancer Vaccine Against Neoantigens Expressed by the Autologous Cancer	Metastatic gastrointestinal cancer	mRNA vaccine encoding neoantigens expressed by the autologous cancer.	I/II	Clinical response not observed. In 3 of the 4 patients a CD8 and CD4+ neoantigen-specific T cells response was detected.
NCT02035956: IVAC MUTANOME Phase I Clinical Trial	Malignant melanoma	mRNA vaccine (personalized, multi-neoepitope “mutanome” vaccine).	I	Well tolerated, no serious adverse events. ~60% of selected neoepitopes induced measurable T-cell responses. Robust CD4⁺ and CD8⁺ T-cell activation observed.
NCT02316457: RNA-Immunotherapy of IVAC_W_bre1_uID and IVAC_M_uID (TNBC-MERIT)	Triple-negative breast cancer	mRNA vaccine: includes two RNA products per patient: a WAREHOUSE (shared TAAs) and a MUTANOME/neoantigen arm.	I	All patients in this trial developed CD4⁺/CD8⁺ T-cell responses directed against 1-10 neoepitopes, with de novo high magnitude responses sustained for about 6 months (10.3% of peripheral CD8⁺ T-cell).
NCT01829971: A Multicenter Phase I Study of MRX34, MicroRNA miR-RX34 Liposomal Injection	Refractory solid tumors: viral-related HCC, melanoma (non-cutaneous, excluding uveal), small cell lung cancer, triple-negative breast cancer, sarcoma, and bladder, renal, and ovarian cancers.	Synthetic, double-stranded miR-34a mimic that is 23-nt in length and encapsulated in a liposomal nanoparticle	I	16 out of 50 patients had clinically significant Stable Disease for ≥4 cycles. The trial was terminated early due to severe immune-mediated toxicities and dead in four patients.
NCT03908671: Clinical Study of Personalized mRNA Vaccine Encoding Neoantigen in Patients With Advanced Esophageal Cancer and Non-small Cell Lung Cancer	Unresectable or metastatic advanced esophageal and non-small cell lung cancers	Personalized mRNA Tumor Vaccine Encoding Neoantigen in Patients		Currently recruiting patients
NCT04573140: A Study of RNA-lipid Particle (RNA-LP) Vaccines for Newly Diagnosed Pediatric High-Grade Gliomas (pHGG) and Adult Glioblastoma (GBM) (PNOC020)	Histopathologically proven newly-diagnosed de novo GBM	Autologous total tumor mRNA and pp65 full length (fl) lysosomal associated membrane protein (LAMP) mRNA loaded DOTAP liposome vaccine	I	Currently recruiting patients

A compelling example of this strategy is the clinical trial NCT03480152, conducted by Rosenberg et al., in which a personalized mRNA vaccine was developed to treat four patients with metastatic gastrointestinal cancer^9^. For the vaccine, named mRNA-4650, tumor samples from each patient were extracted and sequenced to identify somatic mutations capable of generating neoantigens recognizable by the patient’s immune system, specifically by tumor-infiltrating lymphocytes (TILs), which were subsequently isolated and expanded in culture. Following this, high-throughput immunological screening was performed using long peptides and tandem minigenes to determine which neoantigens were effectively recognized by these immune cells. These selected neoantigens, along with mutations in key oncogenes such as *TP53*, *KRAS*, and *PIK3CA*, as well as 15 additional potential neoantigens identified through in silico analysis, were incorporated into the final vaccine formulation^10^. This approach exemplifies how the integration of tumor sequencing, in silico prediction, and functional neoantigen selection enables the transformation of a patient’s individual mutations into highly personalized therapeutic interventions, reinforcing the potential of mRNA-based vaccines as a precision immunotherapy tool.

Currently, no international regulatory agencies have established specific regulations that define a clear framework for the research and validation of mRNA-based cancer vaccines as standard treatments in oncology. Although certain general regulations may apply to these therapies, there is no concrete regulatory guidance that addresses the criteria for evaluating the safety, efficacy, and quality of this type of immunotherapy. For instance, the primary World Health Organization (WHO) guidance document on the topic explicitly limits its scope to “mRNA vaccines for the prevention of infectious diseases”, excluding therapeutic applications like cancer treatments^11^. In particular, the FDA’s Guidance for Industry: Clinical Considerations for Therapeutic Cancer Vaccines^12^ outlines criteria for clinical trials and Investigational New Drug (IND) applications; however, it does not provide specific guidelines regarding the use of mRNA as an immunological adjuvant in oncology. In contrast, the European Medicines Agency (EMA) has adopted a more flexible approach toward advanced therapies, which could serve as a reference for strengthening international regulatory policies^13^.

The absence of a defined regulatory framework poses a significant barrier to the clinical translation of these vaccines, which must overcome multiple challenges. These include demonstrating clinical efficacy through immune responses in patients that correlate with improved survival and tumor control; identifying reliable biomarkers to select patients most likely to respond; and ultimately, the large-scale production of personalized vaccines targeting tumor-specific neoantigens. This last aspect represents a considerable technical and economic challenge, as it requires the development of highly specialized manufacturing platforms with strict quality control standards and optimized production timelines to ensure clinical and commercial viability^14,15^.

Paradoxically, the lack of a specific regulatory framework for mRNA-based cancer vaccines not only poses a challenge to their clinical implementation but also drives the search for innovative strategies and emerging technologies. Although numerous limitations hinder the use of mRNA specifically in cancer immunotherapy, one potential solution lies in exploring alternative therapeutic approaches that constitute a viable line of research while also combating cancer.

In this context, microRNAs (miRNAs) have gained attention as key molecules in gene expression regulation and tumor microenvironment modulation^16^. Their ability to influence critical pathways involved in proliferation, apoptosis, and immune evasion has positioned them as potential adjuvants in immunotherapy, either as modulators of mRNA vaccine-induced responses or as independent therapeutic agents^17^. Integrating miRNAs into RNA-based therapeutic strategies offers a promising approach to optimizing cancer immunotherapy and overcoming some of the current limitations of personalized vaccines.

## microRNAs: from promise to practice

miRNAs are biomolecules classified as non-coding RNAs that regulate gene expression at the post-transcriptional level, playing a key role in various biological processes under normal physiological conditions^18,19^. For instance, miR-1 is a highly conserved miRNA among species and is expressed in muscle tissue, particularly in cardiac muscle, where it is involved in embryonic development of the heart^20^. Similarly, the miR-15/16 family regulates a broad network of genes in T lymphocytes, influencing their differentiation, proliferation, cell cycle restriction, and memory formation^21^. On the other hand, miR-375 plays a crucial role in glucose homeostasis by regulating insulin secretion in pancreatic β-cells through modulation of genes involved in exocytosis and cellular function^22^.

Despite their physiological roles, various miRNAs have been identified as key players in multiple cellular processes associated with cancer development. Within the framework of the hallmarks of cancer, miRNAs have been shown to contribute to proliferative signaling, evasion of tumor suppression and immune destruction, invasion, and metastasis. Significantly, individual miRNAs can modulate multiple oncogenic pathways simultaneously, highlighting their role as integrative regulators of cancer biology^23^.

It has been observed that approximately 50% of these miRNAs are in fragile sites of the genome or in regions prone to deletions and amplifications—events commonly associated with carcinogenesis^19^. The first miRNAs characterized in cancer were miR-15 and miR-16-1 in chronic lymphocytic leukemia (CLL), identified within a deleted region at the 13q14 locus^24^. In most CLL cases, these genes were either absent or expressed at reduced levels. Similarly, other miRNAs are dysregulated in different malignancies, including miR-21, miR-155, and miR-200c in breast cancer^25,26^, as well as miR-141, miR-375, and miR-423-5p in prostate cancer^27^.

Further underscoring this modulatory versatility, miR-375 represents one of the best characterized miRNAs at the mechanistic level, regulating key proteins that control pro-carcinogenic signaling pathways (such as YAP1 and PI3K/AKT), in a tumor-type dependent manner. Notably, in lung cancer, miR-375 has shown to modulate tumor growth, invasion, and therapeutic response through direct regulation of components of these oncogenic pathways^28–30^. In the same way, miR-371 has emerged as a miRNA with a well-defined oncogenic mechanism, particularly in germ cell tumors^31^, where it promotes malignant transformation by regulating transcriptional activity associated with pluripotency and cell cycle-related networks^32,33^.

The dysregulation of miRNA expression not only highlights their central role in carcinogenesis but also positions them as valuable molecular diagnostic tools in clinical practice. Beyond their potential as biomarkers for a more precise molecular classification of tumors—thus improving disease diagnosis and prognosis—their application in immunotherapy presents them as a promising alternative in cancer treatment, opening new avenues for the development of innovative therapeutic strategies^19^. Among the most notable examples is Cobomarsen (MRG-106), a miR-155 inhibitor that has demonstrated preclinical efficacy in models of acute myeloid leukemia (AML) and cutaneous T-cell lymphoma (CTCL), and is currently under clinical evaluation^34,35^. Likewise, the synthetic mimetic MRX34, based on the tumor suppressor miR-34a, was the first miRNA-based therapeutic to enter clinical trials in humans, tested in patients with triple-negative breast cancer, metastatic prostate cancer, and hepatocellular carcinoma^36^. Although the trial was halted due to immune-related adverse events, it initiated a new direction for future development of safer miRNA-based interventions.

Integrating miRNAs into cancer therapeutics is currently being explored through two main approaches: first, harnessing their inhibitory potential to block oncogenic miRNAs (oncomiRs), thereby restoring tumor suppressor gene function; and second, using miRNA mimics to replicate the expression and function of tumor suppressor genes^37^. Ongoing preclinical studies and clinical trials are investigating miRNA replacement therapies, including the use of anti-miR oligonucleotides (AMOs), chemically modified AMOs, and miRNA sponges, among other strategies^38^.

One of the most relevant clinical trials is NCT02369198, which evaluated a miR-16 mimic encapsulated in TargomiRs, nanoparticle-based systems derived from bacteria known as minicells or EnGeneIC Delivery Vehicles (EDVs). These particles were specifically targeted to the EGFR receptor on the surface of mesothelioma cells using bispecific antibodies derived from panitumumab, an anti-EGFR monoclonal antibody. These engineered antibodies were designed to simultaneously bind to EGFR on tumor cells and to a specific antigen on the EDVs, enabling precise and effective delivery of the therapeutic agent^39^.

This intervention aimed to restore the reduced expression of the miR-15/16 family, whose downregulation has been associated with tumor growth in preclinical models of malignant pleural mesothelioma and non-small cell lung cancer (NSCLC). The study showed that a weekly dose of 1.5 μg of RNA encapsulated in 5 × 10⁹ EDVs was well tolerated by patients and exhibited signs of antitumor activity. Among the 22 patients treated, 1 (5%) achieved a partial response lasting 32 weeks, 15 (68%) maintained stable disease, and 6 (27%) experienced disease progression. Additionally, the patient with the partial response showed a significant improvement in lung function, with a 28% increase in forced vital capacity (FVC) and a 20% increase in forced expiratory volume in 1 s (FEV1)^40^. These findings highlight how the therapeutic restoration of dysregulated microRNAs, such as the miR-15/16 family, can elicit measurable clinical benefits, reinforcing the feasibility of RNA-based interventions as a viable strategy for tumor control.

On the other hand, advances in the design and synthesis of delivery systems, structural frameworks, and chemical modifications have driven the development of miRNAs as viable therapeutic tools, bringing them closer to clinical application. Their specificity and mechanism of action support their potential in oncology, where a key strategy involves targeting tumor–type–specific transmembrane receptors. The identification of mutations in these receptors enables the development of highly specific targeted therapies. For instance, in breast cancer, the estrogen receptor (ER), encoded by the *ESR1* gene, may harbor mutations in its ligand-binding domain, making it an attractive target for personalized therapies^41^. Moreover, post-transcriptional regulation of *ESR1* by microRNAs such as miR-206, which directly binds to the 3′UTR of *ESR1* mRNA and suppresses its expression^42^, opens the possibility of therapeutic delivery of miR-206 mimics using lipid nanoparticles (LNPs), an approach already validated for RNA-based medicines in oncology^43^and vaccination^44^, positioning RNA–LNP platforms as a promising strategy for precise modulation of hormone receptor signaling.

**Fig. 1:**
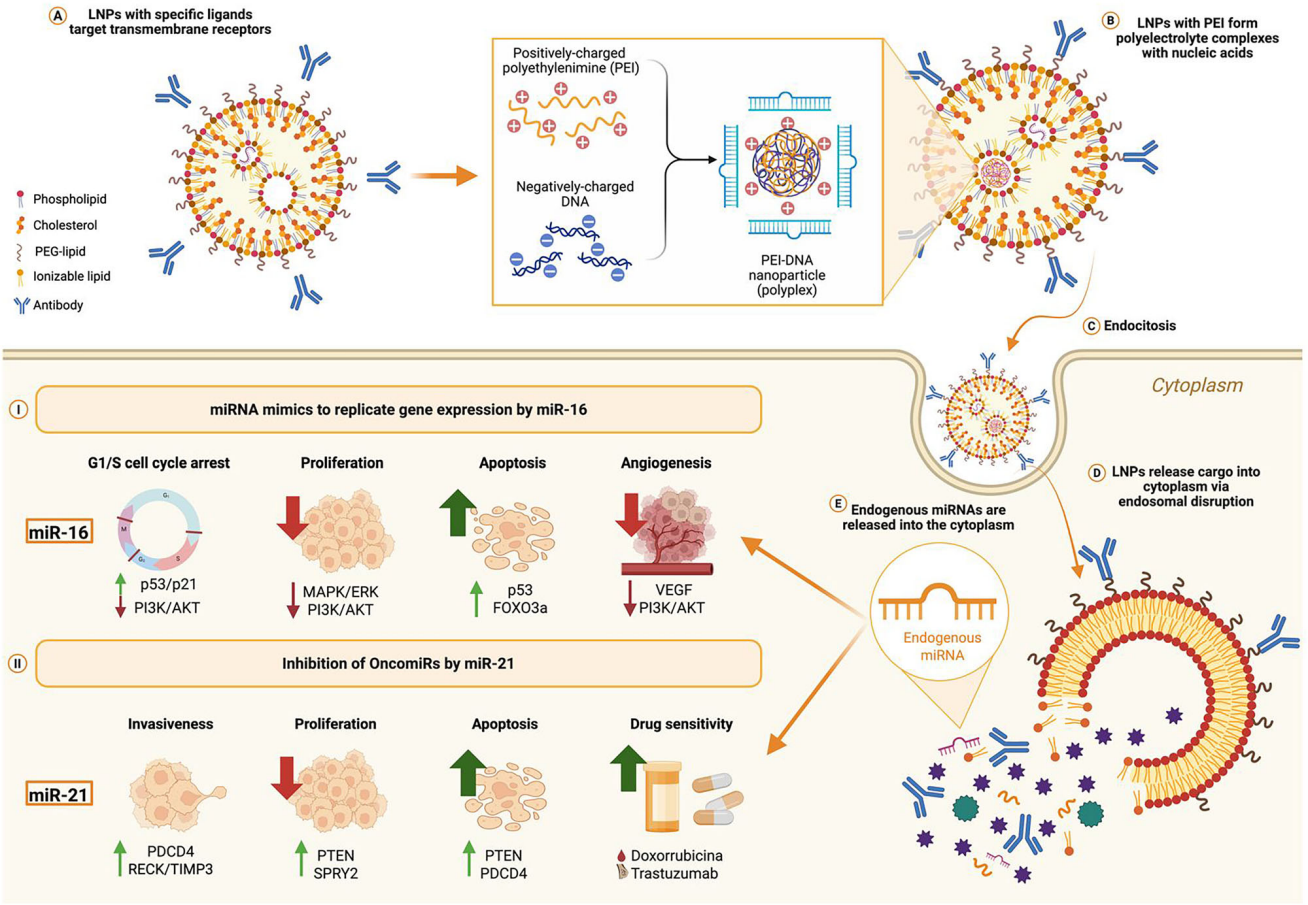
Lipid nanoparticle–based strategies for miRNA delivery in cancer vaccines. Lipid nanoparticles (LNPs) serve as advanced platforms for administering therapeutic miRNAs in cancer immuno-vaccines. **A** LNPs recognize transmembrane receptors. **B** With polymers such as polyethylenimine (PEI), they form polyelectrolyte complexes with nucleic acids (polyplexes). **C** Particles are internalized by endocytosis. **D** Endosomal escape releases the cargo into the cytoplasm. **E** Endogenous miRNAs function as mimics or oncomiR inhibitors—for example, miR-16 (mimic) induces G1/S arrest ( ↑ p53/p21), reduces proliferation ( ↓ MAPK/ERK, PI3K/AKT), increases apoptosis ( ↑ p53, FOXO3a), and limits angiogenesis ( ↓ VEGF/PI3K/AKT), whereas inhibiting the oncomiR miR-21decreases invasiveness ( ↑ PDCD4, RECK/TIMP3), reduces proliferation ( ↑ PTEN, SPRY2), promotes apoptosis ( ↑ PTEN, PDCD4), and enhances drug sensitivity (doxorubicin, trastuzumab). Figure created with BioRender.

A promising strategy involves the use of lipid nanoparticles functionalized with ligands specific to transmembrane receptors, designed to deliver miRNA mimics or inhibitors. These nanoparticles incorporate natural and synthetic polymers capable of forming polyelectrolyte complexes with nucleic acids, such as polyethyleneimine (PEI)^45,46^. Once internalized by cells through endocytosis, the nanoparticles release their cargo into the cytoplasm following endosomal disruption, facilitated by the cationic nature of PEI^46^. In the cytoplasm, miRNAs regulate gene expression by either restoring tumor suppressor gene activity or inhibiting oncomiRs, depending on the cancer’s molecular context (Fig. 1). These advances in nanotechnology open the door to miRNA-based therapies with high precision and therapeutic promise.

Despite their great therapeutic potential, the clinical application of miRNAs still faces significant challenges, including the optimization of delivery systems, the stability of therapeutic miRNAs, accurate dose determination, and the risk of unintended gene regulation, as a single miRNA can modulate multiple targets. For instance, miR-21, one of the most studied oncomiRs, has been shown to influence a wide range of genes involved in apoptosis, cell proliferation, and invasion, including PTEN, PDCD4, and RECK^19^, among many others. Furthermore, although lipid nanoparticles (LNPs) have emerged as one of the most promising delivery platforms for nucleic acid therapeutics, their large-scale production, formulation stability, and potential immunogenicity remain cost-intensive hurdles that limit broader clinical deployment. Therefore, future research must prioritize the development of selective miRNA–LNP formulations with improved tissue specificity, reduced off-target effects, and scalable manufacturing, to fully harness the therapeutic value of miRNAs in oncology.

## Towards a new paradigm in cancer therapeutics

Three decades after their discovery, Victor Ambros and Gary Ruvkun were awarded the Nobel Prize in Medicine for identifying the miRNA lin-4, encoded by the gene of the same name. This miRNA exhibited sequence complementarity to a critical region of the lin-14 mRNA, and together, Ambros and Ruvkun demonstrated that their interaction inhibits the production of the LIN-14 protein in a *C. elegans* model. Their work highlighted the role of miRNAs in post-transcriptional gene regulation and underscored their undeniable impact on biology and medicine^47–49^.

Although there are over 15,000 publications related to miRNAs and cancer in PubMed, and more than 8,000 directly addressing therapeutic applications, to date, no clinical trial has reached phase III or received FDA approval^50^. This contradiction between the vast academic and scientific research and its limited translation into therapeutic applications raises a fundamental question: why does the development of miRNA-based therapies and vaccines for cancer remain so restricted despite the wealth of existing knowledge? Part of the answer lies in our interpretation of the human genome. The coding portion—representing only 1.2% of the genome—has dominated therapeutic development and the biotechnology market, while the remaining 98%, which corresponds to over 20,000 potentially functional genes, remains largely unexplored^51^. This gap can be attributed to technical factors such as low in vivo stability, limitations in preclinical models, and high production costs, all of which slow progress toward advanced clinical phases.

However, the major bottleneck is not discovery—it is clinical translation. Although hundreds of preclinical studies have demonstrated the therapeutic efficacy of miRNA mimics or inhibitors^52^, very few have successfully advanced beyond Phase I or II clinical trials^53^. This stalled progression is largely attributable to low in vivo stability and rapid renal clearance, as native miRNAs are rapidly degraded by serum nucleases and eliminated through renal filtration, necessitating extensive chemical modifications such as 2′-O-methyl, locked nucleic acids (LNA), or phosphorothioate backbones, which increase manufacturing complexity and cost while potentially altering biological activity^54^. In addition, efficient and tissue-specific delivery remains a critical challenge, since miRNAs must reach the cytoplasm of defined cell types, and even delivery strategies based on lipid nanoparticles (LNPs), viral vectors, or exosome-based carriers often show preferential hepatic accumulation rather than effective penetration into solid tumors^44^.

On top of that, their short length and high sequence similarity between family members complicate specific detection and accurate quantification, limiting their robust implementation as diagnostic or prognostic biomarkers. Precazzini and collaborators categorize the main obstacles affecting miRNA-based diagnostics into three major groups^55^: pre-analytical factors, including physiological and pathological variability; analytical factors, related to differences in experimental efficiency and platform performance; and post-analytical factors, associated with standardization and data normalization. Currently, multiple approaches, such as quantitative PCR, next-generation sequencing, and microarray-based analysis, are employed as first-line strategies for miRNA detection, each offering advantages and emphasizing the critical importance of assay selection and detection accuracy^56^.

Another key limitation is off-target activity and gene network complexity, as a single miRNA can regulate dozens or even hundreds of transcripts. While this pleiotropy may enhance therapeutic breadth, it also increases the risk of unintended immunological or metabolic effects, leading to greater scrutiny during clinical evaluation^36^. Moreover, standard murine preclinical models fail to recapitulate critical aspects of human RNA metabolism, extracellular vesicle trafficking, and innate immune sensing, resulting in limited predictability of both efficacy and toxicity during translational assessment^57–59^. Finally, manufacturing and scalability barriers further constrain progress, as GMP-grade synthesis of chemically modified oligonucleotides and their encapsulation in clinically acceptable nanoparticle formulations require specialized infrastructure, driving production costs far beyond those of small molecules or monoclonal antibodies^60^.

Consequently, while miRNAs represent biologically compelling therapeutic entities, their pharmacological development pathway is considerably more complex than that of mRNA vaccines or siRNA-based drugs, which have already achieved regulatory success^61,62^. Overcoming these obstacles will require the integration of rational RNA engineering, targeted delivery platforms, predictive bioinformatic tools to manage off-target interactions, and updated regulatory frameworks tailored to multi-target gene modulators.

## Conclusion and perspectives

In conclusion, advances in the understanding and application of RNA-based therapies have reshaped the landscape of translational medicine, which still reflects a narrow view of the genome’s full therapeutic potential. While mRNA vaccines have proven their feasibility and efficacy in the context of infectious diseases, their application in oncology continues to face regulatory, technical, and economic challenges that hinder clinical implementation. In this context, miRNAs have emerged as a promising alternative in cancer immunotherapy, albeit with their own set of challenges related to stability, delivery, and clinical validation.

Despite these obstacles, the combination of both strategies represents an innovative approach with the potential to revolutionize cancer treatment. The integration of therapeutic RNA technologies, supported by advances in fields such as bioengineering, nanotechnology, and molecular biology, opens the door to novel personalized treatments and the optimization of immune regulation against cancer. Ultimately, to ensure these therapies move “from bench to bedside,” the development of clear regulatory frameworks, robust clinical trials, and strategies that ensure accessibility is essential. Only through a collaborative approach can RNA-based therapeutics be established as a cornerstone of cancer treatment, ushering in a new paradigm in the fight against cancer and the advancement of precision medicine. However, the translation of miRNA-based therapies into late-stage clinical trials remains limited not due to lack of efficacy, but because traditional clinical trial designs—optimized for single-target small molecules—are poorly suited to evaluate multi-target modulators like miRNAs, whose effects unfold across entire gene regulatory networks rather than isolated pathways. Current regulatory endpoints rely on linear cause–effect relationships, whereas miRNAs operate through distributed phenotypic rewiring that may not produce immediate radiographic responses despite inducing durable immunological or metabolic shifts.

To overcome this mismatch, it is proposed that future clinical trials adopt adaptive, systems-biology-driven frameworks integrating longitudinal transcriptomic, proteomic, and immune monitoring rather than relying solely on conventional RECIST outcomes. Furthermore, decentralized “rapid manufacturing hubs” for GMP-grade RNA formulations—leveraging microfluidic-based lipid nanoparticle (LNP) encapsulation—should be explored to reduce batch variability, accelerate production, and enable responsive iteration of miRNA payloads based on real-time patient molecular profiling.

It is proposed to evaluate combined mRNA/miRNA therapies alongside immune checkpoint inhibitors, given their complementary ability to enhance antitumor immune responses. Particular attention should be given to challenges related to scalability, decentralized manufacturing, and economic accessibility in low- and middle-income countries.

## Data Availability

This Perspective does not present original datasets. All information discussed is derived from previously published studies, which are fully cited within the manuscript.
